# Laser capture microdissection of human pancreatic islets reveals novel eQTLs associated with type 2 diabetes

**DOI:** 10.1016/j.molmet.2019.03.004

**Published:** 2019-03-18

**Authors:** Amna Khamis, Mickaël Canouil, Afshan Siddiq, Hutokshi Crouch, Mario Falchi, Manon von Bulow, Florian Ehehalt, Lorella Marselli, Marius Distler, Daniela Richter, Jürgen Weitz, Krister Bokvist, Ioannis Xenarios, Bernard Thorens, Anke M. Schulte, Mark Ibberson, Amelie Bonnefond, Piero Marchetti, Michele Solimena, Philippe Froguel

**Affiliations:** 1 Imperial College London, Department of Genomics of Common Disease, London, UK; 2 University of Lille, CNRS, Institute Pasteur de Lille, UMR 8199 - EGID, F-59000, Lille, France; 3 Sanofi-Aventis Deutschland GmbH, Diabetes Research, Frankfurt, Germany; 4 Department of Visceral-Thoracic-Vascular Surgery, University Hospital Carl Gustav Carus and Faculty of Medicine, TU Dresden, 01307, Dresden, Germany; 5 Paul Langerhans Institute Dresden of the Helmholtz Center Munich at University Hospital Carl Gustav Carus and Faculty of Medicine, TU Dresden, 01307, Dresden, Germany; 6 German Center for Diabetes Research (DZD e.V.), 85764, Neuherberg, Germany; 7 University of Pisa, Department of Clinical and Experimental Medicine, Pisa, Italy; 8 Lilly Research Laboratories, Eli Lilly, 46285-0001, Indianapolis, IN, USA; 9 Vital-IT Group, Swiss Institute of Bioinformatics, 1015, Lausanne, Switzerland; 10 Center for Integrative Genomics, University of Lausanne, Genopode Building, Lausanne, 1015, Switzerland

**Keywords:** Type 2 diabetes, eQTLs, Genetics, Islets, Laser capture microdissection, LCM, Laser Capture Microdissection, PPP, Phenotyped Pancreatectomized Patients

## Abstract

**Objective:**

Genome wide association studies (GWAS) for type 2 diabetes (T2D) have identified genetic *loci* that often localise in non-coding regions of the genome, suggesting gene regulation effects. We combined genetic and transcriptomic analysis from human islets obtained from brain-dead organ donors or surgical patients to detect expression quantitative trait *loci* (eQTLs) and shed light into the regulatory mechanisms of these genes.

**Methods:**

Pancreatic islets were isolated either by laser capture microdissection (LCM) from surgical specimens of 103 metabolically phenotyped pancreatectomized patients (PPP) or by collagenase digestion of pancreas from 100 brain-dead organ donors (OD). Genotyping (> 8.7 million single nucleotide polymorphisms) and expression (> 47,000 transcripts and splice variants) analyses were combined to generate cis-eQTLs.

**Results:**

After applying genome-wide false discovery rate significance thresholds, we identified 1,173 and 1,021 eQTLs in samples of OD and PPP, respectively. Among the strongest eQTLs shared between OD and PPP were *CHURC1* (OD *p*-value=1.71 × 10^-24^; PPP *p*-value = 3.64 × 10^–24^) and *PSPH* (OD *p*-value = 3.92 × 10^−26^; PPP *p*-value = 3.64 × 10^−24^)*.* We identified eQTLs in linkage-disequilibrium with GWAS *loci* T2D and associated traits, including *TTLL6*, *MLX* and *KIF9* loci, which do not implicate the nearest gene. We found in the PPP datasets 11 eQTL genes, which were differentially expressed in T2D and two genes (*CYP4V2* and *TSEN2*) associated with HbA1c but none in the OD samples.

**Conclusions:**

eQTL analysis of LCM islets from PPP led us to identify novel genes which had not been previously linked to islet biology and T2D. The understanding gained from eQTL approaches, especially using surgical samples of living patients, provides a more accurate 3-dimensional representation than those from genetic studies alone.

## Highlights

• We identified eQTLs from human islets: 1,173 eQTLs from organ donors and 1,021 from surgical samples.
• eQTLs in LD with GWAS *loci* for T2D and associated traits did not always implicate the nearest gene.
• We identified 11 eQTL genes differentially expressed in T2D compared to controls only in PPP surgical patients.
• Two genes (*CYP4V2* and *TSEN2*) associated with elevated HbA1C only in PPP.

## 1 Introduction

Genome-wide association studies (GWAS) meta-analyses have revealed >200 *loci* associated with type 2 diabetes (T2D) and associated traits, such as fasting glucose [1], [2], [3], [4], [5]. Recently, new statistical methods such as fine-mapping have identified further 40 *loci* [6]. However, deciphering the causal variants and making inferences from GWAS to physiology is still a challenge. Firstly, few GWAS single nucleotide polymorphisms (SNPs) are near insightful biological candidate genes. More importantly, most SNPs fall within the non-coding, regulatory regions of the genome [7], often far from coding regions or in regions where more than one gene lies. To overcome these complexities, combining GWAS with expression data to produce expression quantitative trait loci (eQTLs) has become a powerful tool to shed light into the causative mechanisms of these genetic associations [6], [8], [9], [10]. However, one limitation is that whilst there is a considerable overlap across human tissue transcriptomes, many eQTLs are cell type specific. Therefore, it is crucial to analyse eQTLs from appropriate tissues, e.g. those functionally involved in the pathology. With regard to diabetes, only a few studies have investigated eQTLs in pancreatic islet samples [11], [12]. However, an insufficient supply of human islets, and technical issues related to the origin of the samples (i.e. from brain-dead organ donors rather than from surgical living patients), have restricted the performance of such studies. Here, we tested a total of 203 islet samples from European subjects with the aim to identify islet cis-eQTLs related to T2D.

## 2 Materials and methods

### 2.1 Cohort

Blood and pancreatic tissue samples were collected from a total of 203 patients from two independent cohorts. The first cohort comprised of 100 islet samples isolated by limited proteolytic digestion of pancreas from brain-dead organ donors (OD), which included 19 subjects with T2D [13]. The second cohort consisted of 103 islet samples isolated by laser capture microdissection (LCM) from the healthy margins of surgical fragments of metabolically phenotyped pancreatectomized patients (PPP), who underwent surgery for different pancreatic diseases [13]. In the PPP cohort, 32 were normoglycaemic (fasting glycaemia <7.0 mmol/l; HbA1C ≤ 6.5%, glycaemia at 2 h after presurgical oral glucose tolerance test [OGTT] <7.8 mmol/l), 15 had impaired glucose tolerance (IGT, fasting glycaemia <7.0 mmol/l; HbA1C ≤ 6.5%, OGTT at 2 h of ≥7.8 to <11.1 mmol/l), 36 had T2D (fasting glycaemia ≥7.0 mmol/l; HbA1C >6.5%, history of diabetes for >1 year) and 20 had type 3 diabetes (T3D), based on clinical history and pre-surgical laboratory tests according to the American Diabetes Association (ADA) guidelines. T3D was defined as a secondary form of diabetes with an onset not longer than 1 year prior to the appearance of the symptoms related to the primary pancreatic disease. Samples were collected as part of the IMIDIA consortium, and more information on the donors in these cohorts has been recently reported [13].

### 2.2 Islet collection and RNA extraction

Specific details about the methodologies for islet retrieval and RNA extraction have been previously described [13]. Briefly, RNA was extracted from OD islet samples with the PicoPure RNA Isolation Kit, following the manufacturer's protocol. Islets were extracted using collagenase extraction methods [14]. Islets of PPP were retrieved from cryopreserved surgical samples by LCM with a Zeiss Palm MicroBeam system. RNA was extracted using the Arcturus PicoPure Isolation kit.

### 2.3 Expression microarray

RNA was analysed for gene expression profiling using the Affymetrix Human Genome U133 Plus 2.0 Array (deposited under the accession number GSE76896), according to the manufacturer's protocol. Data from Gene Expression Omnibus (GEO) and annotation data have been imported using R package affy. The array data include 54,210 probe sets covering over 47,000 transcripts and splice variants. Expression data was normalised using the Robust Multichip Analysis (RMA) method as implemented in R package affy [15]. Possible batch effect was corrected for using the “ComBat” approach implement in R package sva [16], using only the batch information. Differential expression analysis to compare non-diabetic versus T2D samples was performed using Limma (Bioconductor R package) with age and gender as covariates. Multiple testing was accounted for using Benjamini-Hochberg method to correct p-values. PPP and OD samples clustered separately (Supplementary Fig. 4a), but there was no difference in clustering in other factors, such as gender (Supplementary Fig. 4b). For PPP, as the HbA1C levels were similar between ND controls and IGT, both samples were categorised as controls and compared to T3D and T2D samples.

### 2.4 DNA extraction and genotyping

**Figure 1:**
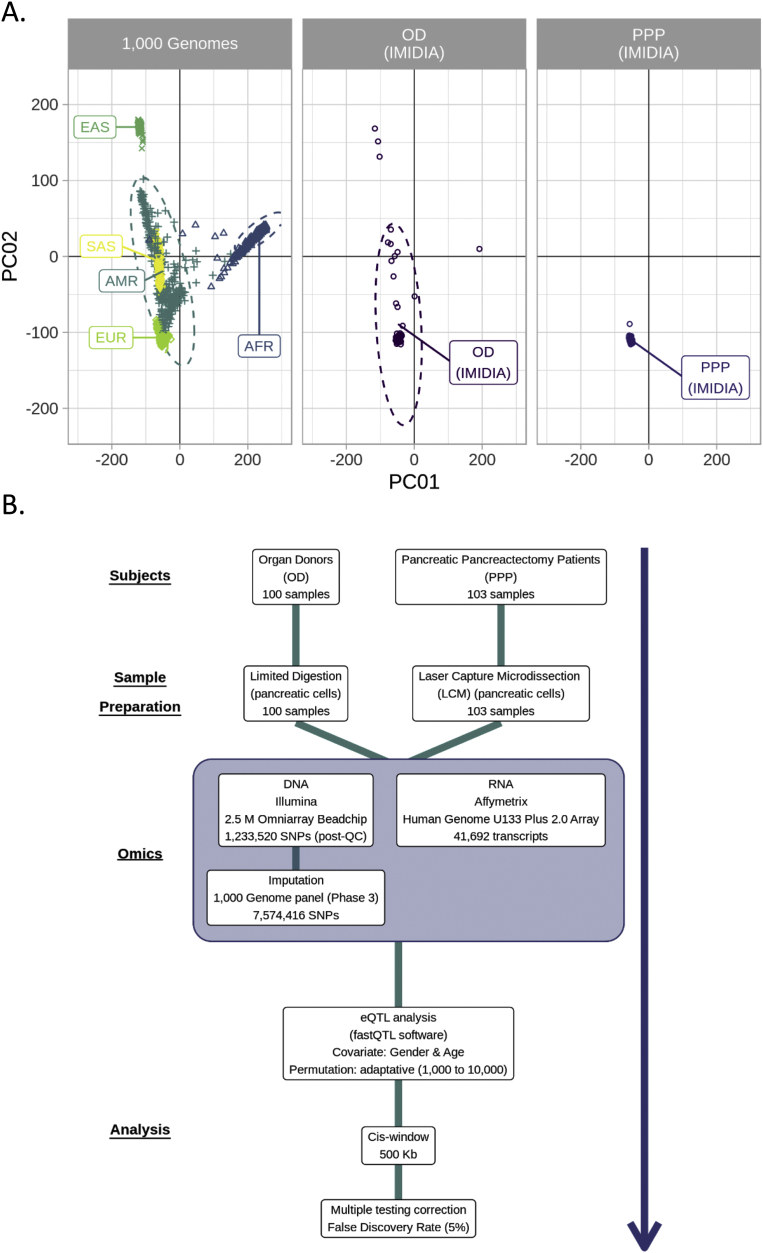
**Ancestry clustering and eQTL methodology. A.** Genotype clustering of samples from PPP and OD samples confirmed European descent of subjects, compared to the 1,000 genomes. **B.** An overview of the methodology utilized to obtain eQTLs from islets of OD and PPP subjects were isolated by limited digestion from OD material and by LCM from PPP material. Acis-window of 500 kb was used with adjustment on gender and age and a false discovery rate (FDR) of <5% was used as a cut-off.

A total of 203 DNA samples were extracted from either blood or pancreatic tissue and isolated using the DNeasy Blood & Tissue kit (Qiagen, Germany). All samples were genotyped using the Illumina Omni2.5M array and run on the Illumina iScan platform at the Imperial College London Centre (Hammersmith Campus, Imperial College London, UK). Genotypes were called using the Genome-studio software. Quality-control was performed and SNPs were kept for further analysis according to the following thresholds: 1/maf >0.05, 2/Hardy-Weinberg equilibrium >0.001 and 3/call rate >0.95. Prephasing was performed with ShapeIt (v2.r790) and imputation with Impute2 (v2.3.2) using 1,000 genomes panel (phase 3). This lead to a dataset of 1,233,520 SNPs and imputation lead to a further 7,574,416 SNPs, for a total of >8.7 million SNPs analysed. Principal component analysis (PCA) for ethnicity using genomics data from 1,000 genomes confirmed the vast majority of samples were of European descent (Figure 1A).

### 2.5 eQTL analysis

eQTL analysis was carried out on all 203 samples. The eQTL analysis was performed using FastQTL software [17], with gender and age as covariates. *P*-values were computed using an adaptive permutation pass approach with a permutation number set from 1,000 to 10,000. We performed a genome-wide genotyping and expression analysis in OD and PPP samples to identify eQTLs (Figure 1B). At first, a cis-window was defined as 1 Mb between SNPs and expression probes and then reduced to 500 Kb based on the observed *p*-values distribution (Supplementary Fig. 2). All eQTLs with a FDR<5% were considered to be significant. The eQTL regions were annotated to published binding sites of human islet transcription factors [18], which were subdivided into five classes: C1 (promoters), C2 (inactive enhancers), C3 (active enhancers), C4 (CTCF-bound sites) and C5 (others), which was not CTCF-bound and was not bound to a known histone modification.

### 2.6 Bioinformatics

The Database for Annotation, Visualisation and Integrated Discovery (DAVID) is a software tool that clusters proteins into functional annotations to facilitate understanding of complex datasets [19] (https://david.ncifcrf.gov/). The DAVID tool was used to cluster genes to identify enriched gene ontological functions. *P*-values were calculated using Fisher's exact test to score the enriched gene ontology terms. In addition, the OD and PPP eQTL datasets were exported to Ingenuity Pathways Analysis (IPA) Software (Ingenuity Systems, Incorporated, California, USA; www.ingenuity.com). A core analysis was performed to identify pathways. The pathways were organised according to significance using a Fisher's exact test, with a cut-off of a –log (p-value) < 1.3. A z-score was used to predict downstream regulator activity (activation or inhibition) patterns. For the upstream analysis, a *p*-value < 0.01 was used for relationships between regulators.

## 3 Results

### 3.1 Identification of cis-eQTLs in human islet samples from two distinct cohorts

**Figure 2:**
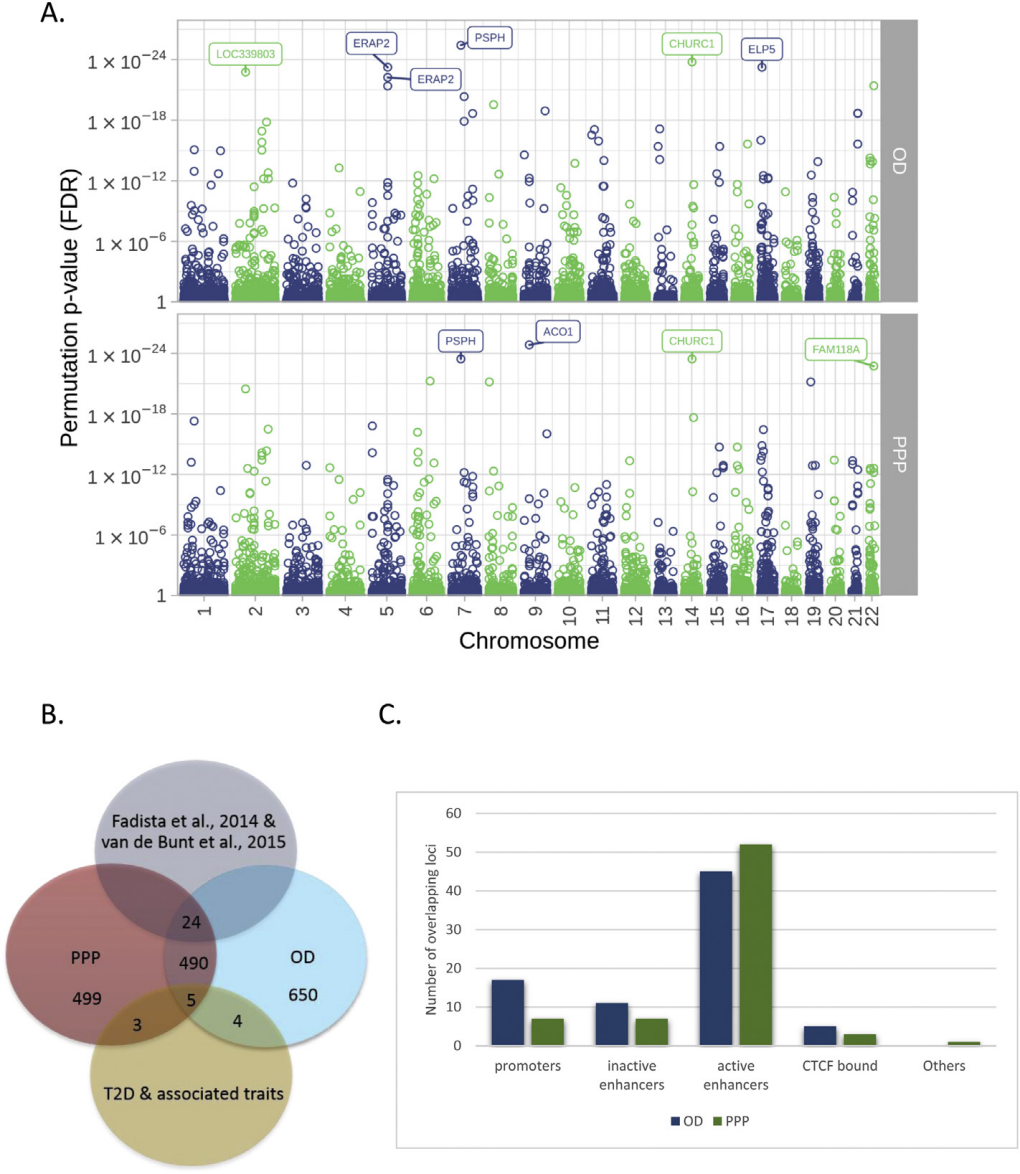
**Overview of eQTL significant *loci*. A.** Manhattan plot of the significant cis-eQTLs (≤500 kb), showing the top 10 significant eQTLs (FDR<5%), from 103 PPP and 100 OD subjects. **B.** Venn diagram summarising number of genes shared between PPP, OD, GWAS and previously identified eQTL genes in islets (Fadista et al., 2014; van de Bunt et al., 2015). **C.** An overview of the number of eQTL locations within putative regulatory regions within the genome that correspond with eQTL regions in OD and PPP datasets.

Our analysis resulted in the identification of a total of 1,173 and 1,021 significant eQTLs in the samples of OD and PPP, respectively (Supplementary Table 1). We found that only 60% of eQTL genes were shared between the two cohorts and were consistent in the direction of the effect. These differences could conceivably be attributed to differences in the method for islet isolation between the two cohorts. Among the strongest eQTLs shared between OD and PPP were *CHURC1* (OD *p*-value = 1.71 × 10^−24^; PPP *p*-value = 3.64 × 10^−24^) and *PSPH* (OD *p*-value = 3.92 × 10^−26^; PPP *p*-value = 3.64 × 10^−24^) (Figure 2A). Churchill domain containing 1 (*CHURC1*) eQTLs have recently been reported to be associated with T2D and obesity in both adipose and muscle tissues [20], [21], suggesting a pleiotropic effect of this gene in T2D pathophysiology. Phosphoserine phosphatase (*PSPH*) catalyses the last step of serine biosynthesis from carbohydrates [22]. Other strong eQTLs unique in either dataset were *ACO1* in PPP (*p* = 1.47 × 10^−25^) and *ELP5* (*p*-value = 5.71 × 10^−24^) in OD samples. Aconitase 1 (ACO1) is an essential enzyme involved in the TCA cycle and cellular iron homeostasis [23]. ELP5 is a member of the RNA polymerase II elongator complex, which plays a role in chromatin remodelling and histone acetylation [24].

Of the identified eQTLs in OD and PPP, 42 were transcription factors. This was determined using the TFcheckpoint, a resource for transcription factors that provides a comprehensive list of transcription factor annotations with evidence from the literature [25]. In addition, a total of 46 in OD and 46 in PPP non-coding RNAs, including long non-coding and non-protein coding RNAs, were found to be significantly affected by nearby SNPs. Of these, 26 were shared between the OD and PPP datasets.

We compared our analysis to published eQTLs in islets from OD: one study in 89 OD identified 616 eQTLs [11]; another study in 118 OD found 2,341 eQTLs [12]. While the reproducibility rate between the two studies was 43% [12], we found a 28% (OD) and 29% (PPP) shared genes with van de Bunt et al. [12] and 23% (OD) and 18% (PPP) with Fadista et al. [11]. Of these, 24 genes were shared between our OD and PPP samples and those in these previous datasets, consistent in direction of effect (Table 1; Figure 2B). This low number is likely due to substantial differences in RNA extraction and transcriptomic methods among these studies. For instance, we used microarrays for transcriptomic analysis, whereas the other studies relied on RNA sequencing. Nevertheless, 42% of the shared genes were among the top 100 identified eQTLs in our study, including *CHURC1, ERAP2*, and *LDHC.*

**Table 1:** A list of 24 genes shared among OD, PPP Fadista et al., 2014 and Van de Bunt et al., 2015.

Gene	Gene Name	OD p-value	PPP p value
CHURC1	Churchill Domain Containing 1	1.7E-24	3.6E-24
ERAP2	Endoplasmic Reticulum Aminopeptidase 2	5.8E-23	5.6E-12
FAM118A	Family With Sequence Similarity 118 Member A	3.9E-22	1.9E-23
POMZP3	POM121 And ZP3 Fusion	4.7E-21	6.4E-13
LDHC	Lactate Dehydrogenase C	8.6E-18	1.7E-10
C17orf97	Chromosome 17 Open Reading Frame 97	9.8E-17	4.0E-14
UBE2U	Ubiquitin Conjugating Enzyme E2 U (Putative)	8.5E-16	9.9E-10
ITGB3BP	Integrin Subunit Beta 3 Binding Protein	1.2E-13	5.2E-18
ZFP57	ZFP57 Zinc Finger Protein	7.6E-11	6.6E-17
DDX11	DEAD/H-Box Helicase 11	2.1E-10	4.5E-14
THNSL2	Threonine Synthase Like 2	1.5E-08	6.5E-11
TDRD5	Tudor Domain Containing 5	3.3E-08	6.0E-06

### 3.2 Regulatory and biological interpretations of eQTL genes

Consistent with these regions being regulatory, we annotated the eQTL datasets with active open chromatin maps in islets (promoters, active enhancers and CTCF regions) [15] and found 102 and 93 sites overlapping with open chromatin regions in OD and PPP, respectively (Figure 2C, Supplementary Tables 2–3). Of these, 15 were shared between the two datasets, with the majority being in active enhancer sites and clusters. One shared gene relevant for beta-cell biology was *RPH3AL/NOC2*, which has been shown to be crucial for exocytosis of insulin in pancreatic beta cells [26].

**Figure 3:**
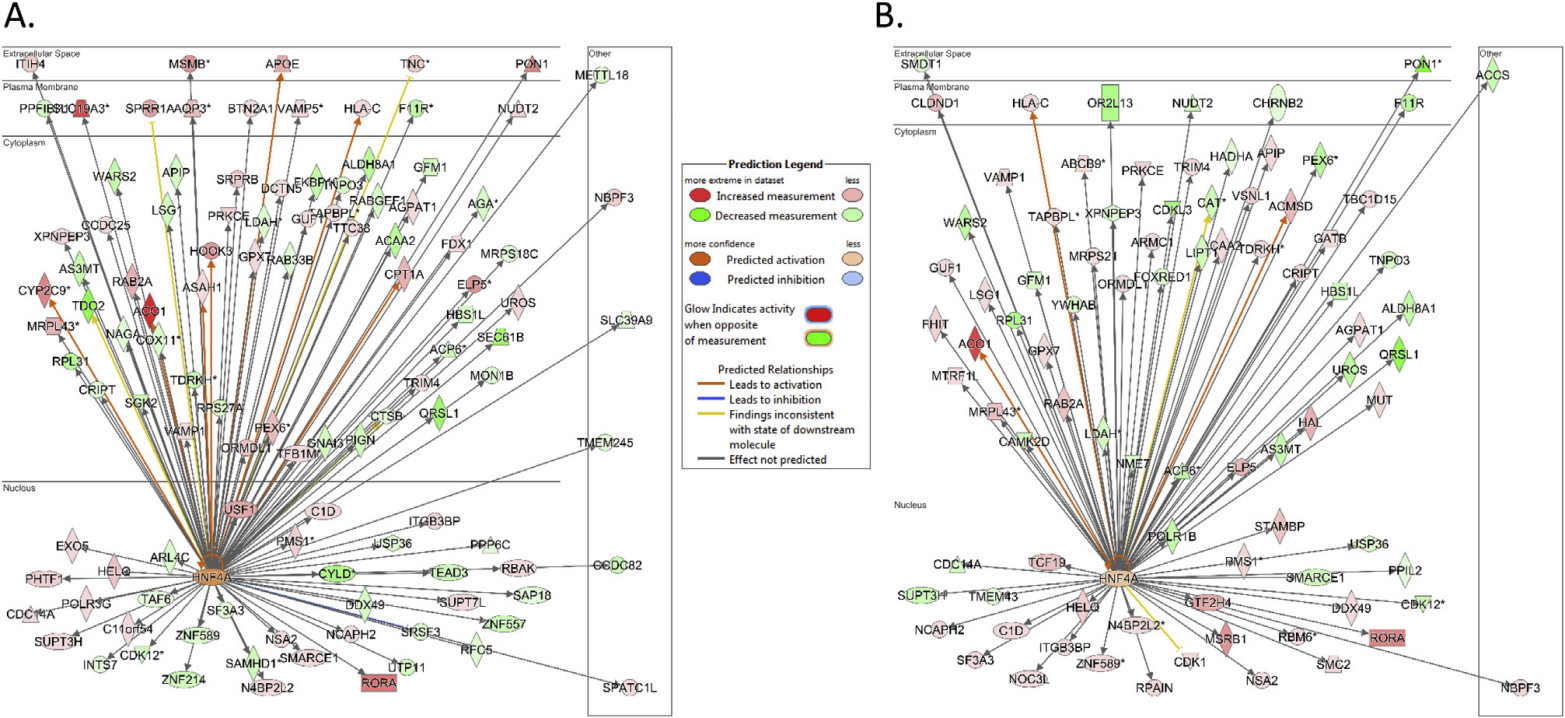
**Biological interpretations of eQTL genes.** Genes in our dataset that have been shown with IPA to have the MODY gene *HNF4A* as the upstream regulator for **A.** OD and **B.** PPP. Genes highlighted in purple have a relevant function in carbohydrate metabolism, lipid metabolism or diabetes.

The gene ontology (GO) tool was used to make inferences on the biological function of the eQTL genes. GO identified ontologies that relate to beta cell function included cell junction, positive regulation of GTPase activity and glutathione metabolism (Supplementary Fig. 3). In addition, to make inferences about the identified eQTL genes, we utilised tools in the Ingenuity Pathway Analysis (IPA) software. None of the top pathways was shared between OD and PPP samples, pointing to differences in the donors and the procedure for islet isolation (Supplementary Fig. 4). Interestingly, the transcriptional regulator of pancreatic beta-cell function and maturity-onset diabetes of the young (MODY4) gene *HNF4A* was found to the most significant upstream regulator in the IPA analysis for both PPP (*p*-value = 0.003; z-score = 0.45) and OD (*p*-value = 0.0001; z-score = 0.98) (Figure 3). HNF4A modulates regulatory elements in the promoters and enhancers of genes involved in glucose, fatty acid and cholesterol metabolism. It regulates gene expression in pancreatic beta cells to achieve glucose homeostasis and activates insulin genes both directly and indirectly. Our data show that many genes have a relevance for carbohydrate and lipid metabolism in addition to their association with T2D.

### 3.3 eQTL genes associated with T2D and associated traits

We then focused our analysis on GWAS *loci* and genes for T2D and associated traits. First, we found an enrichment for GWAS *loci* in eQTLs in MAGIC fasting glucose (OD *p* = 4.8 × 10^−4^; PPP *p* = 4.1 × 10^−4^), fasting insulin (OD *p* = 0.013; PPP *p* = 0.059), HOMA-IR (OD *p* = 0.019; PPP *p* = 0.018) and HOMA-B (OD *p* = 0.001; PPP *p* = 0.0025) and DIAGRAM (OD *p* = 0.0039; PPP *p* = 3.7 × 10^−4^) using a Fisher's exact test (Supplementary Table 4). We then investigated whether T2D genes were also identified in our eQTL study and found a total of eleven genes (Table 2). Seven of these overlapped GWAS genes associated with T2D alone (*AP3S2, GPSM1, MAEA, TLE4, TMEM163, SSR1*, *and UBE2Z*), three with T2D associated traits alone (*IGF1R* for fasting plasma glucose, *ABO* for disposition index and *UHRF1BP1* for fasting plasma insulin), and one for both T2D and fasting glucose levels (*ADCY5*). Of these genes, *AP3S2* and *IGF1R* were represented in both our OD and PPP datasets. *AP3S2*, encoding for an adaptor protein, was the only gene replicated in both our datasets in addition to reported eQTLs in islets [11], [12]. ADCY5 is a member of the adenylate cyclase family, which has previously been shown in a knockout study to be involved in glucose stimulation and insulin secretion [27]. Interestingly, we have previously demonstrated that *SSR1* expression is enriched in human islets and isolated beta cells [28]. In addition, for both OD and PPP datasets, we found strong associations with *UBE2Z*, a ubiquitin-conjugating enzyme. Although this gene has not been previously been studied for its role in beta cells, *UBE2Z* was also reported to be associated with coronary artery disease in different populations [29], [30], [31]. *UHRF1BP1* was the only gene that was annotated to an active enhancer in pancreatic islets. We and others from the GIANT, MAGIC and ExomeBP consortia, recently identified 40 additional *loci* associated with T2D [6] and found that *TPCN2*, a gene encoding for a transmembrane ion channel with an effect on insulin action, also has an eQTL in both PPP and OD. *TPCN2*^*−/−*^ mice display improved insulin sensitivity, which is in agreement with our findings that an increased expression of *TPCN2* associated with the rs72928978 reported SNP [6].

**Table 2:** GWAS T2D and glycaemic genes identified in eQTL study.

Gene	Gene Name	Disease/Trait	PPP	OD	Fadista et al., 2014	Van de Bunt et al., 2015
ABO	ABO, Alpha 1-3-N-Acetylgalactosaminyltransferase And Alpha 1-3-Galactosyltransferase	Disposition index and soluble E-selectin levels	Y	Y	Y	
ADCY5	Adenylate Cyclase 5	Type 2 diabetes; fasting glucose-related traits	Y			Y
AP3S2	Adaptor Related Protein Complex 3 Sigma 2 Subunit	Type 2 diabetes	Y	Y	Y	Y
GPSM1	G Protein Signaling Modulator 1	Type 2 diabetes	Y			
IGF1R	Insulin Like Growth Factor 1 Receptor	Fasting plasma glucose	Y	Y		
MAEA	Macrophage Erythroblast Attacher	Type 2 diabetes	Y			
SSR1	Signal Sequence Receptor Subunit 1	Type 2 diabetes		Y		
TLE4	Transducin Like Enhancer Of Split 4	Type 2 diabetes		Y		
TPCN2	Two Pore Segment Channel 2	Type 2 diabetes	Y	Y		Y
TMEM163	Transmembrane Protein 163	Type 2 diabetes		Y		Y
UBE2Z	Ubiquitin Conjugating Enzyme E2 Z	Type 2 diabetes	Y	Y		
UHRF1BP1	UHRF1 Binding Protein 1	Fasting insulin-related traits		Y		

Y=Yes

We then assessed whether our eQTL SNPs were in linkage disequilibrium with GWAS reported SNPs for T2D and associated traits using the GWAS catalogue ((https://www.ebi.ac.uk/gwas/) and a recently published GWAS [6]. The most significant *loci* in the PPP and OD datasets are shown in Table 3. Notably, approximately 60% did not implicate the nearest gene. This includes the *CENTD2* (*ARAP1*) locus, which a recent study confirmed to be associated with T2D risk through the reduced expression of the nearby *STARD10* gene [32]. In addition, one of the most significant eQTLs we found was the rs34569841 SNP, which is associated with decreased expression of the long non-coding RNA LOC101927636. This SNP is in strong linkage disequilibrium with the previously reported rs13134327 SNP (R^2^ = 1), which is associated with HbA1C and was mapped to the *FREM3* gene [33].

**Table 3:** Top GWAS T2D and associated traits loci co-localising with eQTLs.

GWAS Catalog						
GWAS gene	SNPS	eQTL SNP	eQTL distance	eQTL FDR	eQTL beta	Probe ID

PPP						

FREM3	rs13134327	rs5015757	−174187	9.2E-15	−0.96	LOC101927636
UBE2Z	rs12453394	rs318092	1381	1.2E-14	−0.93	UBE2Z
HLA-DQA1	rs9271774	rs9271770	48612	6.7E-11	1.2	LOC100996809

OD						
SSR1	rs9505118	rs3087986	1363	2.6E-12	0.35	SSR1
UBE2Z	rs12453394	rs3744608	3813	4.1E-12	−0.77	UBE2Z
BRAF	rs9648716	rs28529157	81058	2E-08	−0.45	BRAF

Mahajan et al., 2018						

GWAS gene	rs ID	eQTL SNP	eQTL distance	eQTL FDR	eQTL beta	Probe ID

PPP						

TTLL6	rs2032844	rs11657371	−145547	1.8E-05	−0.64	UBE2Z
MACF1	rs2296172	rs61779279	287263	0.0010	−0.2	MACF1
MLX	rs665268	rs646123	−114000	0.0014	0.24	CNTNAP1

OD						
KIF9	rs2276853	rs2276854	−47481	0.0009	−0.32	KLHL18
CENTD2	rs56200889	rs12575364	−56695	0.0009	0.29	STARD10
TTLL6	rs2032844	rs11657371	−145547	0.0014	−0.41	UBE2Z

### 3.4 eQTL genes differentially expressed with T2D and HbA1C levels

Our eQTL analysis further identified in the PPP dataset only 21 genes (Table 4), which were also differentially expressed, 11 of which also consistently in their directionality. Among them was *SCTR,* which encodes for the secretin receptor and is the most potent regulator for bicarbonate in the pancreas. Although early studies showed that SCTR is linked to insulin release in humans (Lerner and Porte 1970), this association has not since been re-investigated. eQTLs in the mitochondrial Acid Phosphatase 6 Lysophosphatidic *ACP6* have been associated with T2D and BMI [34]. Three *loci*, namely *KLHDC10, VAMP1*, and *CFLAR* lie within active islet enhancer regions. Among them, however, only *KLHDC10,* which is involved in the activation of oxidative stress [35], was consistent in its directional effect between eQTL and differential expression. In addition, of the differentially expressed loci, only two were transcription factors (*ZNF117* and *ONECUT2*). Lastly, to identify genes whose expression was altered by glycaemia, we correlated gene expression to HbA1C levels, which is a measure of long-term glycaemia in the PPP dataset. This revealed 170 differentially expressed genes (Supplementary Table 5), of which only four had an eQTL: *CYP4V2* (cytochrome P450 4V2)*, TSEN2* (tRNA-splicing endonuclease subunit 2)*, ZNF556* (zinc finger protein 556) and *ACVR1B* (activin A receptor type 1B), of which *CYP4V2* and *TSEN2* were consistent in directional effect.

**Table 4:** Significant eQTL genes also differentially expressed in T2D compared to controls in PPP dataset.

PPP						
Gene	SNP ID	distance	eQTL FDR	logFC	FDR	Direction of eQTL effect
SCTR	rs4344946	−15760	0.023	−0.78	0.03	–
CYP2U1	rs141713207	−22383	0.002	−0.55	0.01	+
CYP4V2	rs10866290	757	6.38E-11	−0.5	0.05	–
CASR	rs3749203	489	0.001	−0.45	0.04	+
ACP6	rs28700004	5788	7.94E-08	−0.41	0.01	–
LOC79160	rs3761133	653	0.015	−0.39	0.03	+
IGF1R	rs12591122	305650	3.08E-05	−0.34	0.04	+
LINC00667	rs34361006	49303	0.032	−0.31	0.02	–
ACVR1B	rs2854464	43389	0.008	−0.28	0.02	+
KLHDC10	rs4443587	68107	1.58E-10	−0.25	0.01	–
VAMP1	rs11613996	22444	0.016	−0.25	0.02	+
ELP5	rs222843	−9867	1.40E-15	−0.25	0.03	+
RASA3	rs9525230	80306	4.37E-07	−0.22	0.04	+
H2AFV	rs13245012	−669	0.002	−0.22	0.04	–
METTL15	rs4614434	−52250	0.027	−0.15	0.05	–
RNF213	rs35627722	15051	0.01	0.44	0.01	–
CAST	kgp11684924	12272	4.62E-07	0.46	0.02	+
ERAP1	kgp3909205	15984	3.09E-12	0.49	0.03	+
ZNF117	rs10262238	121749	0.015	0.5	0.02	+
CFLAR	rs10184098	9878	0.001	0.63	0.03	–
ONECUT2	rs514250	32118	0.008	0.71	0.04	+

## 4 Discussion

**Figure 4:**
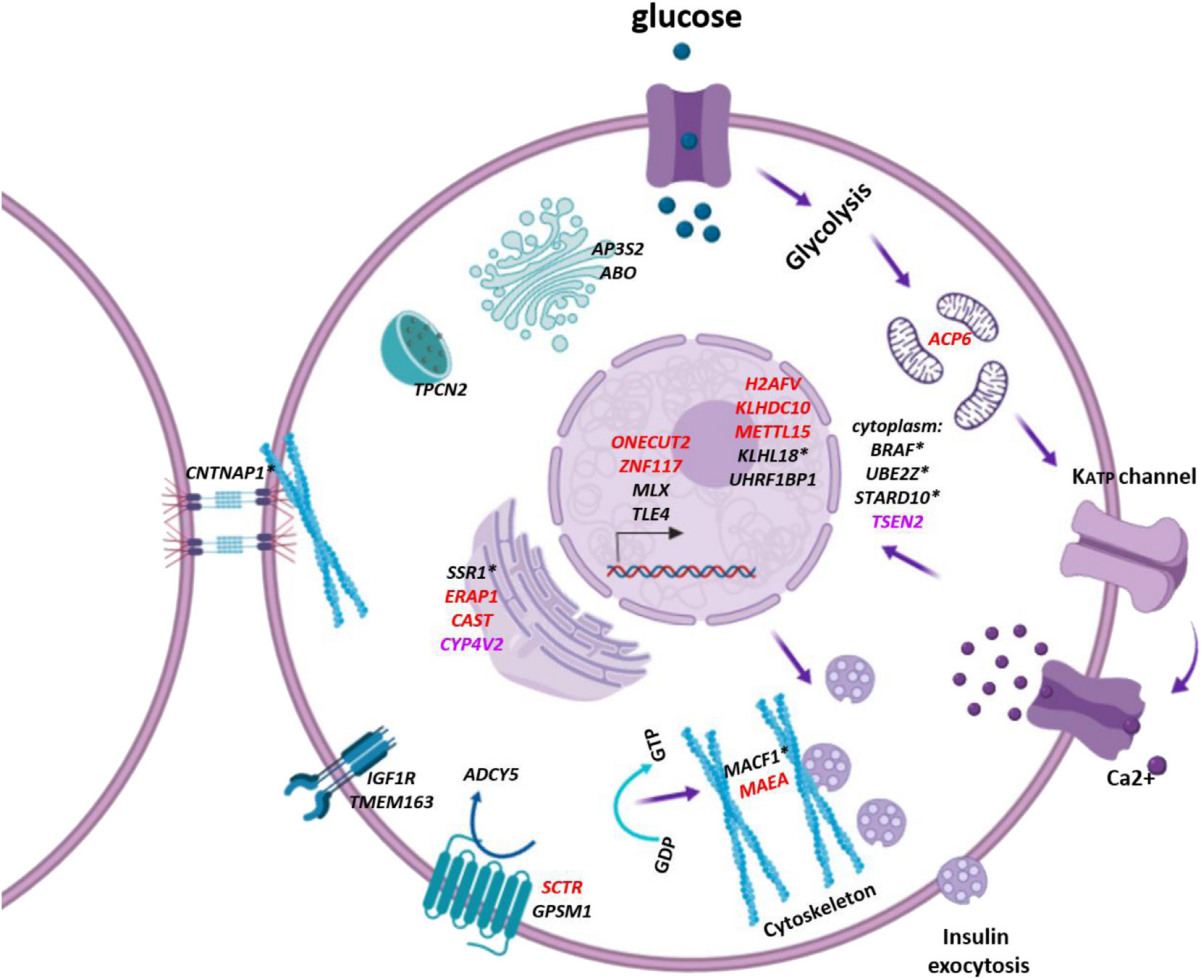
**Summary of eQTL genes with relevance to T2D.** A schematic of a representative pancreatic beta cell, with identified genes annotated to their known sub-cellular localization using gene ontology. These genes include overlapping GWAS genes (black), eQTLs in LD with GWAS *loci* (asterisks), eQTL genes from the PPP dataset that were differentially expressed in T2D (red) or associated with HbA1c (pink), consistent in directional effect with eQTLs. (Figure was illustrated using app.biorender.io).

Here we show the most comprehensive cis-eQTL analysis in relevant islet samples in two distinct cohorts, including samples from 100 OD and for the first time 103 PPP (e.g. from living surgical patients). To our knowledge, this is also the largest eQTL study using islet samples to date. Our thorough cohorts allowed us to identify eQTLs with a relevance to T2D and HbA1C levels. These genes have diverse roles within the cell and further studies need to be performed to understand their roles within the pancreatic beta cell (Figure 4). One main finding was the difference in results observed between our two cohorts. We found that the PPP and OD samples cluster separately, suggesting that the differences in transcriptomic signatures between the two cohorts is primarily due in part to the islet isolation methods. Indeed, we recently compared the transcriptomes of PPP and OD islets and found that those of islets extracted from the same ODs using either LCM or enzymatic digestion clustered separately, with LCM islet transcriptomes clustering with the transcriptomes of LCM islets from PPP [13]. These differences were also highlighted in our pathway analysis using IPA. In addition, these differences may be attributed to stressful conditions while OD patients were in intensive-care prior to brain-death, which may increase inflammatory markers. In contrast, for LCM samples, the surgical specimen are subject to immediate cryofixation, thereby limiting transcriptomic changes. As all previous eQTL and transcriptomic investigations have been performed using islets isolated by enzymatic digestion from OD [11], [12], [36], our study including LCM islets from PPP is unique. In addition to isolation methods, methodological differences in RNA expression analyses likely contribute to the low number of shared genes between our study and previous eQTL analyses in islets [11], [12]. The reason for the use of microarrays in our study is that at the time these studies were conceived and undertaken, RNA-seq procedures (based on next-generation sequencing) were still very expensive and not yet fully consolidated. However, one robust gene that survived these technical and methodological differences and which was replicated amongst all studies is *ZFP57*. Loss-of-function mutations in the *ZFP57* transcription factor is associated with an imprinting disorder, which includes transient neonatal diabetes [37], [38]. *ZFP57* is expressed in undifferentiated cells and downregulated during cell differentiation [39] and is a crucial regulator involved in DNA methylation during development [40]. It would be interesting to further investigate whether DNA methylation changes within this gene has a role in the cell biology of adult beta cells.

In our comparison of GWAS *loci* and eQTLs, we were able to provide an up-to-date analysis of approximately 300 identified genes linked to T2D and associated traits. An intriguing finding was that long non-coding RNAs were found to be the most significantly associated with GWAS SNPs, which includes the long non-coding RNA LOC101927636 *locus*, which in GWAS studies was previously assigned to *FREM3* [33]. This is in addition to approximately 66 non-coding RNAs identified between the OD and PPP datasets. Accumulating literature has shown that SNPs in long non-coding RNAs are associated with human diseases, including T2D, hence highlighting their role as master regulators [41], [42].

It is important to note that islets include a number of cell-types and although we have considered normalising to housekeeping genes identified from single-cell transcriptomic analysis in islets [43], [44], these genes were highly variable among our samples. While it is important to bear in mind that some of the eQTLs identified are not beta-cell specific, there is substantial data to gain from studying the islet as an intact microorgan.

In conclusion, we provide a catalogue of cis-eQTLs from the largest to-date sample size from two separate cohorts of non-diabetic and T2D subjects and which includes the first in-depth combined genetic and expression analysis of human islets isolated by LCM. Ultimately, the knowledge gained from eQTL approaches provides a preferable and more accurate 3-dimensional representation than those from DNA variants alone. *In vitro* and functional analyses are required to definitively prove the role of newly identified genes in relation to islet cell biology and T2D disease.

## References

[1] Voight B.F., Scott L.J., Steinthorsdottir V., Morris A.P., Dina C., Welch R.P. (2010 Jul). Twelve type 2 diabetes susceptibility loci identified through large-scale association analysis. Nature Genetics.

[2] DIAbetes Genetics Replication And Meta-analysis (DIAGRAM) Consortium, Asian Genetic Epidemiology Network Type 2 Diabetes (AGEN-T2D) Consortium, South Asian Type 2 Diabetes (SAT2D) Consortium, Mexican American Type 2 Diabetes (MAT2D) Consortium, Type 2 Diabetes Genetic Exploration by Nex-generation sequencing in muylti-Ethnic Samples (T2D-GENES) Consortium, Mahajan A. (2014 Mar). Genome-wide trans-ancestry meta-analysis provides insight into the genetic architecture of type 2 diabetes susceptibility. Nature Genetics.

[3] Scott R.A., Scott L.J., Mägi R., Marullo L., Gaulton K.J., Kaakinen M. (2017 Nov). An expanded genome-wide association study of type 2 diabetes in Europeans. Diabetes.

[4] Dupuis J., Langenberg C., Prokopenko I., Saxena R., Soranzo N., Jackson A.U. (2010 Feb). New genetic loci implicated in fasting glucose homeostasis and their impact on type 2 diabetes risk. Nature Genetics.

[5] Ingelsson E., McCarthy M.I. (2018 Jun). Human genetics of obesity and type 2 diabetes mellitus: past, present, and future. Circulation: Genomic and Precision Medicine.

[6] Mahajan A., Wessel J., Willems S.M., Zhao W., Robertson N.R., Chu A.Y. (2018 Apr). Refining the accuracy of validated target identification through coding variant fine-mapping in type 2 diabetes. Nature Genetics.

[7] Varshney A., Scott L.J., Welch R.P., Erdos M.R., Chines P.S., Narisu N. (2017 28). Genetic regulatory signatures underlying islet gene expression and type 2 diabetes. Proceedings of the National Academy of Sciences of the United States of America.

[8] Li Q., Seo J.-H., Stranger B., McKenna A., Pe’er I., LaFramboise T. (2013 Jan 31). A novel eQTL-based analysis reveals the biology of breast cancer risk loci. Cell.

[9] Adriaens M.E., Bezzina C.R. (2018 Aug 1). Genomic approaches for the elucidation of genes and gene networks underlying cardiovascular traits. Biophysical Reviews.

[10] Croteau-Chonka D.C., Rogers A.J., Raj T., McGeachie M.J., Qiu W., Ziniti J.P. (2015). Expression Quantitative Trait Loci Information Improves Predictive Modeling of Disease Relevance of Non-Coding Genetic Variation. PLoS One.

[11] Fadista J., Vikman P., Laakso E.O., Mollet I.G., Esguerra J.L., Taneera J. (2014 Sep 23). Global genomic and transcriptomic analysis of human pancreatic islets reveals novel genes influencing glucose metabolism. Proceedings of the National Academy of Sciences of the United States of America.

[12] van de Bunt M., Manning Fox J.E., Dai X., Barrett A., Grey C., Li L. (2015 Dec 1). Transcript expression data from human islets links regulatory signals from genome-wide association studies for type 2 diabetes and glycemic traits to their downstream effectors. PLoS Genetics.

[13] Solimena M., Schulte A.M., Marselli L., Ehehalt F., Richter D., Kleeberg M. (2018). Systems biology of the IMIDIA biobank from organ donors and pancreatectomised patients defines a novel transcriptomic signature of islets from individuals with type 2 diabetes. Diabetologia.

[14] Marchetti P., Bugliani M., Lupi R., Marselli L., Masini M., Boggi U. (2007 Dec). The endoplasmic reticulum in pancreatic beta cells of type 2 diabetes patients. Diabetologia.

[15] Gautier L., Cope L., Bolstad B.M., Irizarry R.A. (2004 Feb 12). affy–analysis of Affymetrix GeneChip data at the probe level. Bioinformatics.

[16] Leek J.T., Johnson W.E., Parker H.S., Jaffe A.E., Storey J.D. (2012 Mar 15). The sva package for removing batch effects and other unwanted variation in high-throughput experiments. Bioinformatics.

[17] Ongen H., Buil A., Brown A.A., Dermitzakis E.T., Delaneau O. (2016 May 15). Fast and efficient QTL mapper for thousands of molecular phenotypes. Bioinformatics.

[18] Pasquali L., Gaulton K.J., Rodríguez-Seguí S.A., Mularoni L., Miguel-Escalada I., Akerman İ. (2014 Feb). Pancreatic islet enhancer clusters enriched in type 2 diabetes risk-associated variants. Nature Genetics.

[19] Huang D.W., Sherman B.T., Lempicki R.A. (2009 Jan). Systematic and integrative analysis of large gene lists using DAVID bioinformatics resources. Nature Protocols.

[20] Sajuthi S.P., Sharma N.K., Chou J.W., Palmer N.D., McWilliams D.R., Beal J. (2016 Aug). Mapping adipose and muscle tissue expression quantitative trait loci in African Americans to identify genes for type 2 diabetes and obesity. Human Genetics.

[21] Glastonbury C.A., Viñuela A., Buil A., Halldorsson G.H., Thorleifsson G., Helgason H. (2016 Sep 1). Adiposity-Dependent Regulatory Effects on Multi-tissue Transcriptomes. The American Journal of Human Genetics.

[22] Sun L., Song L., Wan Q., Wu G., Li X., Wang Y. (2015 Apr). cMyc-mediated activation of serine biosynthesis pathway is critical for cancer progression under nutrient deprivation conditions. Cell Research.

[23] Moreno M., Ortega F., Xifra G., Ricart W., Fernández-Real J.M., Moreno-Navarrete J.M. (2015 Apr). Cytosolic aconitase activity sustains adipogenic capacity of adipose tissue connecting iron metabolism and adipogenesis. FASEB.

[24] Belalcazar L.M., Papandonatos G.D., McCaffery J.M., Peter I., Pajewski N.M., Erar B. (2015 Jun). A common variant in the CLDN7/ELP5 locus predicts adiponectin change with lifestyle intervention and improved fitness in obese individuals with diabetes. Physiological Genomics.

[25] Chawla K., Tripathi S., Thommesen L., Lægreid A., Kuiper M. (2013 Oct 1). TFcheckpoint: a curated compendium of specific DNA-binding RNA polymerase II transcription factors. Bioinformatics.

[26] Matsunaga K., Taoka M., Isobe T., Izumi T. (2017 01). Rab2a and Rab27a cooperatively regulate the transition from granule maturation to exocytosis through the dual effector Noc2. Journal of Cell Science.

[27] Hodson D.J., Mitchell R.K., Marselli L., Pullen T.J., Gimeno Brias S., Semplici F. (2014 Sep). ADCY5 couples glucose to insulin secretion in human islets. Diabetes.

[28] Ndiaye F.K., Ortalli A., Canouil M., Huyvaert M., Salazar-Cardozo C., Lecoeur C. (2017 Apr 8). Expression and functional assessment of candidate type 2 diabetes susceptibility genes identify four new genes contributing to human insulin secretion. Molecular Metabolism.

[29] Guo Y., Wang F., Li L., Gao H., Arckacki S., Wang I.Z. (2017 Jul 14). Genome-wide linkage analysis of large multiple multigenerational families identifies novel genetic loci for coronary artery disease. Scientific Reports.

[30] Bastami M., Ghaderian S.M., Omrani M.D., Mirfakhraie R., Nariman-Saleh-Fam Z., Mansoori Y. (2015 Nov 20). Evaluating the association of common UBE2Z variants with coronary artery disease in an Iranian population. Cellular and Molecular Biology.

[31] Lu D., Huang J., Ma X., Gu N., Zhang J., Zhang H. (2017). Rs46522 in the Ubiquitin-conjugating enzyme E2Z gene is associated with the risk of coronary artery disease in individuals of chinese han population with type 2 diabetes. Journal of Diabetes Research.

[32] Carrat G.R., Hu M., Nguyen-Tu M.-S., Chabosseau P., Gaulton K.J., van de Bunt M. (2017 Feb 2). Decreased STARD10 expression is associated with defective insulin secretion in humans and mice. The American Journal of Human Genetics.

[33] Wheeler E., Leong A., Liu C.-T., Hivert M.-F., Strawbridge R.J., Podmore C. (2017 Sep 12). Impact of common genetic determinants of Hemoglobin A1c on type 2 diabetes risk and diagnosis in ancestrally diverse populations: A transethnic genome-wide meta-analysis. PLoS Medicine.

[34] Sun, Jain R., Andersson L.E., Medina A., Storm P., Spegel P. (2017). Perturbed mitochondrial metabolism in islets from donors with type-2 diabetes. bioRxiv.

[35] Sekine Y., Hatanaka R., Watanabe T., Sono N., Iemura S., Natsume T. (2012 Dec 14). The Kelch repeat protein KLHDC10 regulates oxidative stress-induced ASK1 activation by suppressing PP5. Molecular Cell.

[36] Locke J.M., Hysenaj G., Wood A.R., Weedon M.N., Harries L.W. (2015 Apr). Targeted allelic expression profiling in human islets identifies cis-regulatory effects for multiple variants identified by type 2 diabetes genome-wide association studies. Diabetes.

[37] Boonen S.E., Mackay D.J.G., Hahnemann J.M.D., Docherty L., Grønskov K., Lehmann A. (2013 Mar). Transient neonatal diabetes, ZFP57, and hypomethylation of multiple imprinted loci: a detailed follow-up. Diabetes Care.

[38] Bak M., Boonen S.E., Dahl C., Hahnemann J.M.D., Mackay D.J.D.G., Tümer Z. (2016 Apr 14). Genome-wide DNA methylation analysis of transient neonatal diabetes type 1 patients with mutations in ZFP57. BMC Medical Genetics.

[39] Akagi T., Usuda M., Matsuda T., Ko M.S.H., Niwa H., Asano M. (2005 May 27). Identification of Zfp-57 as a downstream molecule of STAT3 and Oct-3/4 in embryonic stem cells. Biochemical and Biophysical Research Communications.

[40] Quenneville S., Verde G., Corsinotti A., Kapopoulou A., Jakobsson J., Offner S. (2011 Nov 4). In embryonic stem cells, ZFP57/KAP1 recognize a methylated hexanucleotide to affect chromatin and DNA methylation of imprinting control regions. Molecular Cell.

[41] Jain S., Thakkar N., Chhatai J., Pal Bhadra M., Bhadra U. (2017 04). Long non-coding RNA: Functional agent for disease traits. RNA Biology.

[42] Goyal N., Kesharwani D., Datta M. (2018 May 1). Lnc-ing non-coding RNAs with metabolism and diabetes: roles of lncRNAs. Cellular and Molecular Life Sciences.

[43] Segerstolpe Å., Palasantza A., Eliasson P., Andersson E.-M., Andréasson A.-C., Sun X. (2016 Oct 11). Single-cell transcriptome profiling of human pancreatic islets in health and type 2 diabetes. Cell Metabolism.

[44] Muraro M.J., Dharmadhikari G., Grün D., Groen N., Dielen T., Jansen E. (2016 26). A single-cell transcriptome atlas of the human pancreas. Cell Systems.

