# Supplementary material for: Laser capture microdissection of human pancreatic islets reveals novel eQTLs associated with type 2 diabetes
Source: Mol Metab. 2019 Mar 18;24:98–107. doi: 10.1016/j.molmet.2019.03.004 (PMC6531807; doi:10.1016/j.molmet.2019.03.004)
Supplement: Multimedia component 1 [file mmc1.pptx]

## Slide 1
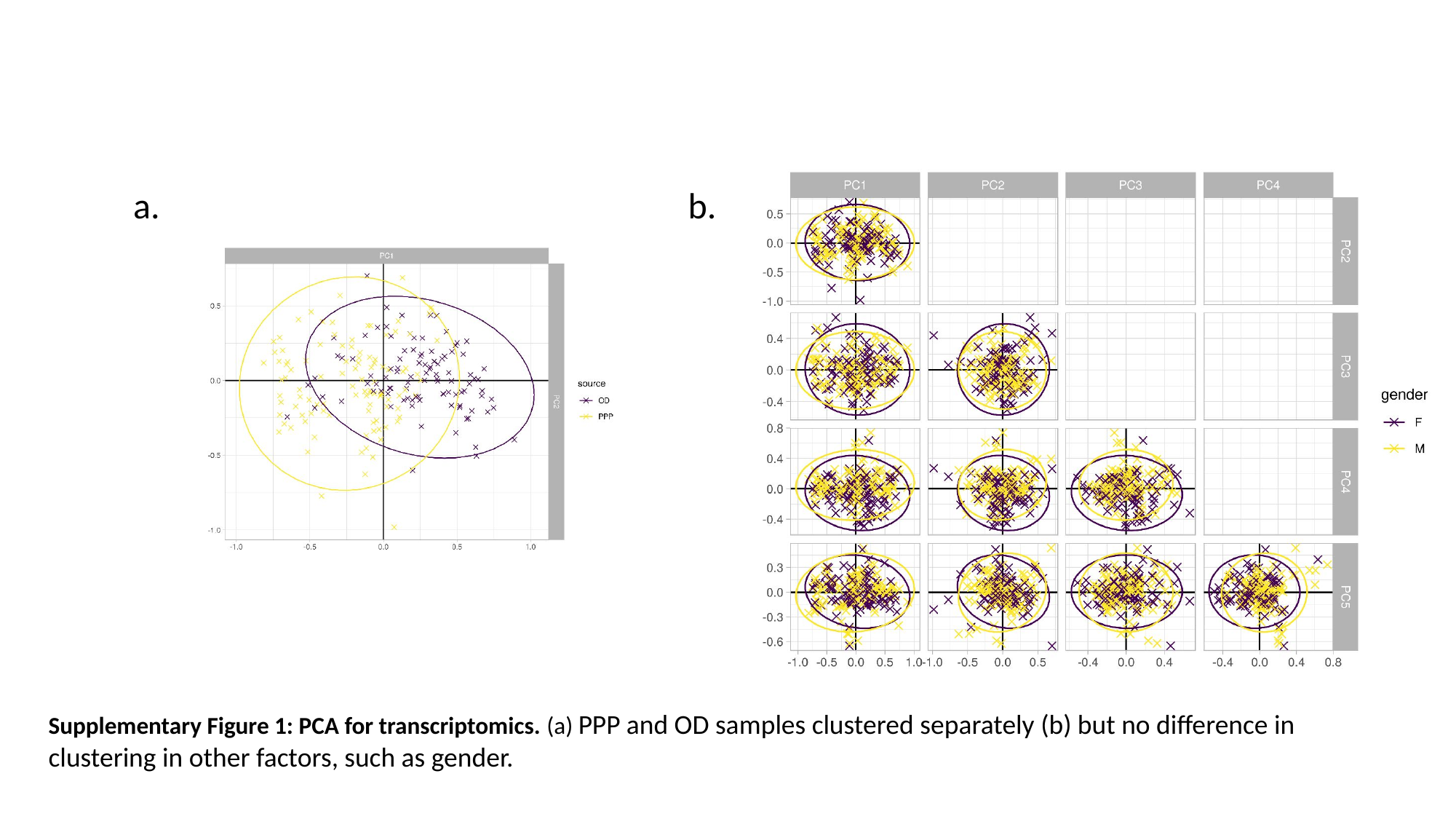

a.
b.
Supplementary Figure 1: PCA for transcriptomics. (a) PPP and OD samples clustered separately (b) but no difference in clustering in other factors, such as gender.

## Slide 2
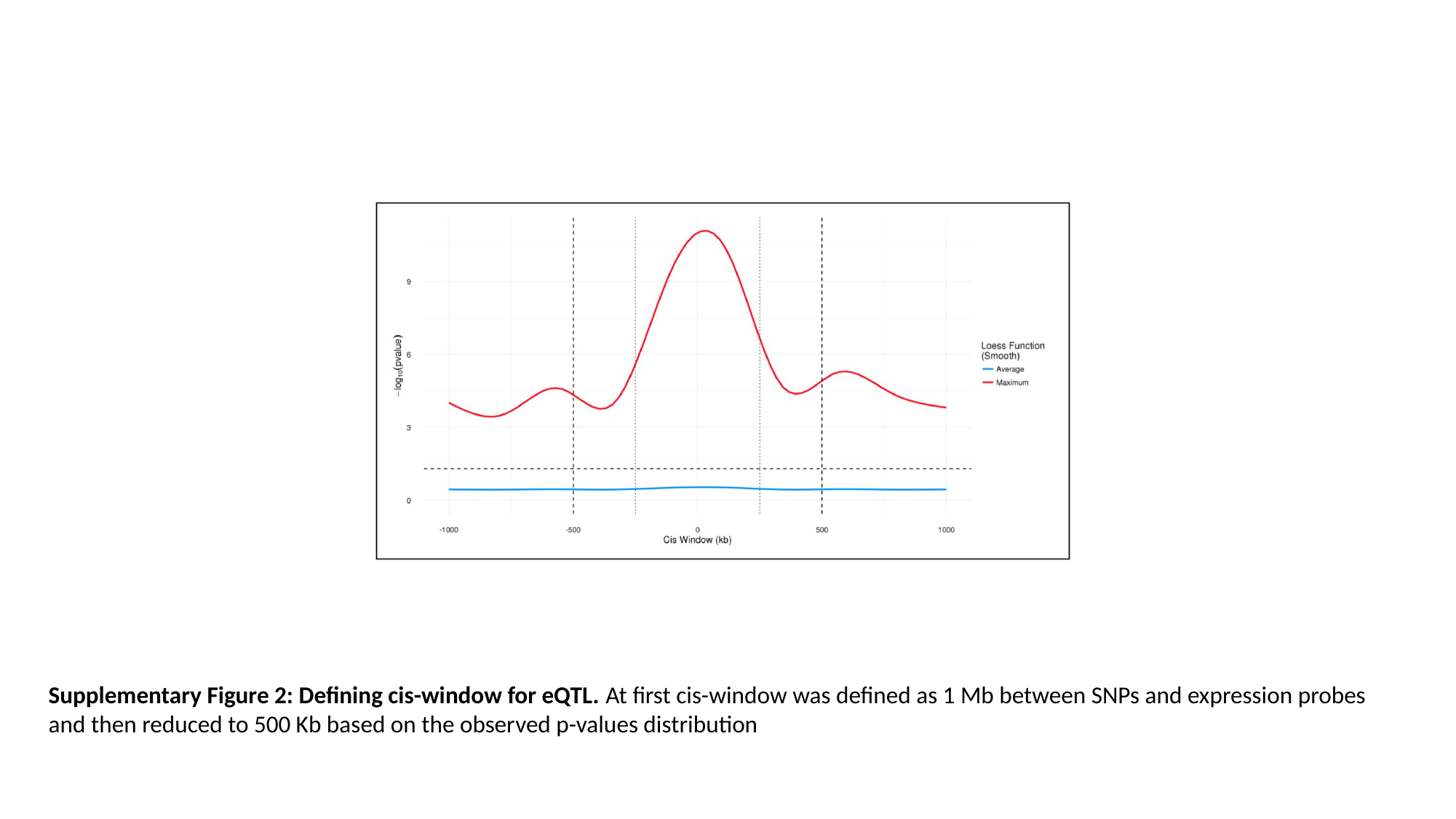

Supplementary Figure 2: Defining cis-window for eQTL. At first cis-window was defined as 1 Mb between SNPs and expression probes and then reduced to 500 Kb based on the observed p-values distribution

## Slide 3
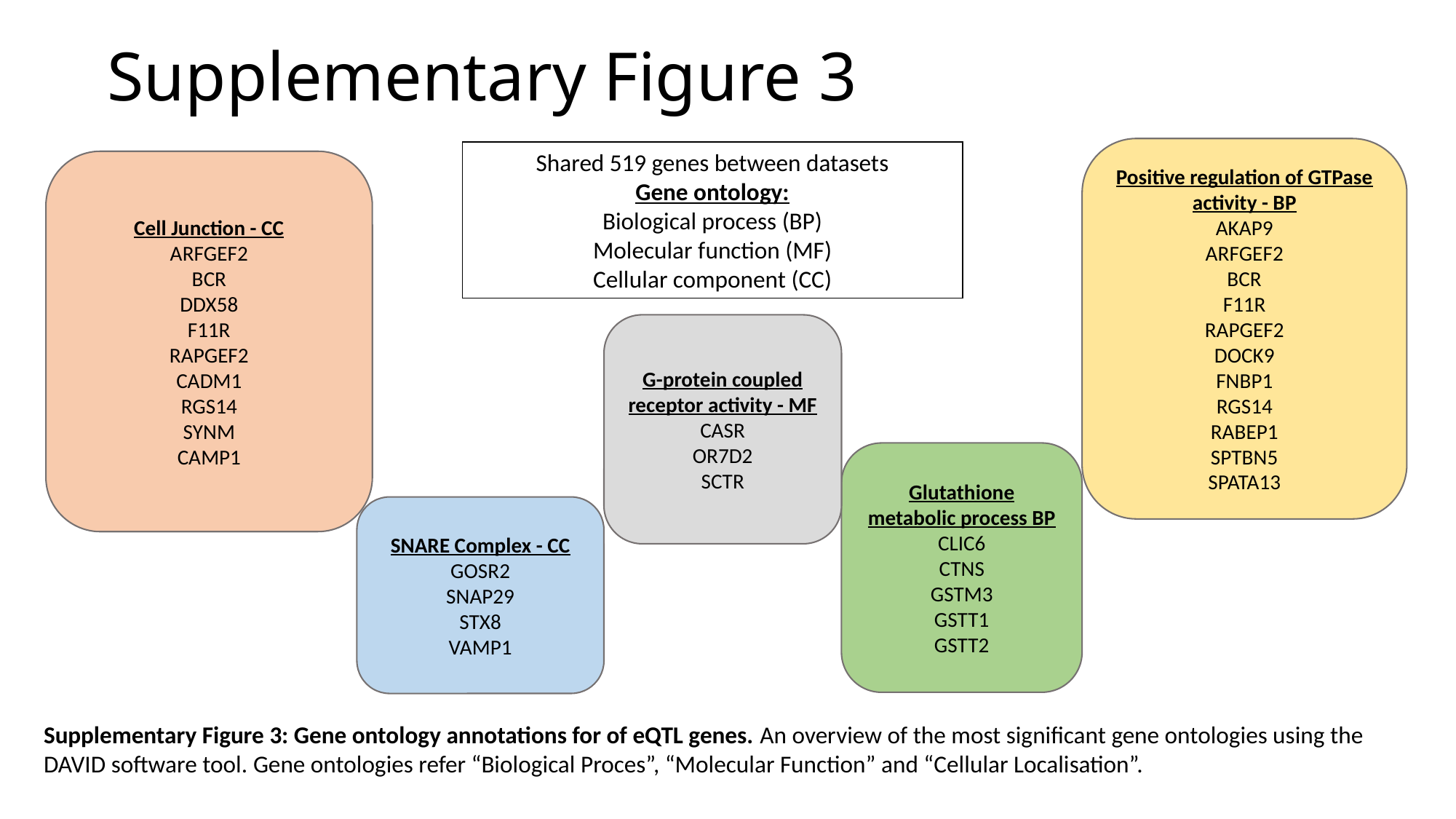

# Supplementary Figure 3
Positive regulation of GTPase activity - BP
AKAP9
ARFGEF2
BCR
F11R
RAPGEF2
DOCK9
FNBP1
RGS14
RABEP1
SPTBN5
SPATA13
Shared 519 genes between datasets
Gene ontology:
Biological process (BP)
Molecular function (MF)
Cellular component (CC)
Cell Junction - CC
ARFGEF2
BCR
DDX58
F11R
RAPGEF2
CADM1
RGS14
SYNM
CAMP1
G-protein coupled receptor activity - MF
CASR
OR7D2
SCTR
Glutathione metabolic process BP
CLIC6
CTNS
GSTM3
GSTT1
GSTT2
SNARE Complex - CC
GOSR2
SNAP29
STX8
VAMP1
Supplementary Figure 3: Gene ontology annotations for of eQTL genes. An overview of the most significant gene ontologies using the DAVID software tool. Gene ontologies refer “Biological Proces”, “Molecular Function” and “Cellular Localisation”.

## Slide 4
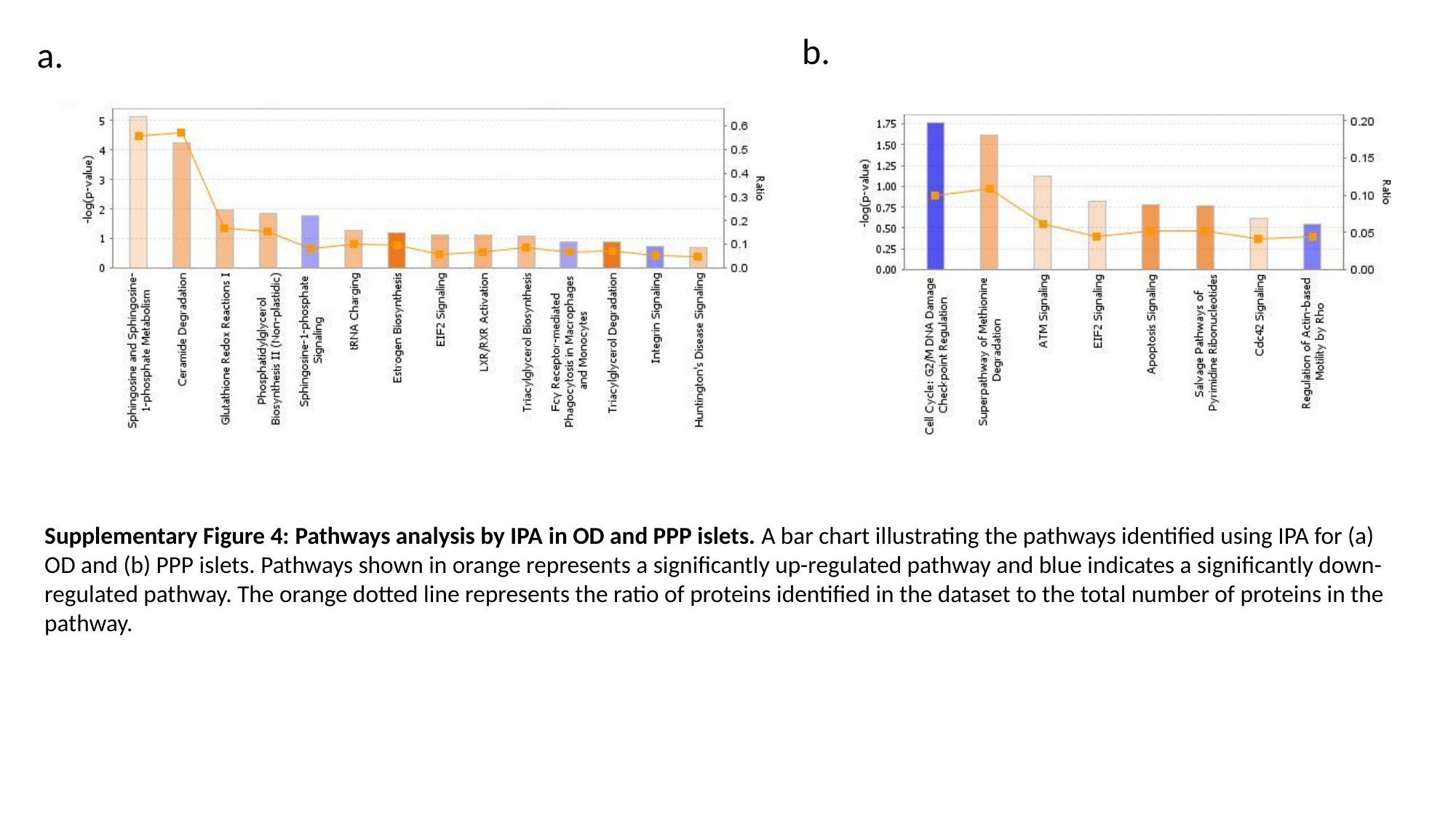

b.
a.
Supplementary Figure 4: Pathways analysis by IPA in OD and PPP islets. A bar chart illustrating the pathways identified using IPA for (a) OD and (b) PPP islets. Pathways shown in orange represents a significantly up-regulated pathway and blue indicates a significantly down-regulated pathway. The orange dotted line represents the ratio of proteins identified in the dataset to the total number of proteins in the pathway.
